# Application of the protection motivation theory in predicting wild mushroom consumption among university students in China

**DOI:** 10.1371/journal.pone.0335719

**Published:** 2025-11-04

**Authors:** Si Chen, Yu Chen, Zhenyi Li

**Affiliations:** 1 China National Center for Food Safety Risk Assessment, Beijing, China; 2 School of Art and Communication, Fujian Polytechnic Normal University, Fuqing, China; 3 Department of Communication and Culture, Royal Roads University, Victoria, Britosh Columbia, Canada; Federal University Otuoke, NIGERIA

## Abstract

**Background:**

Wild mushroom poisoning represents a significant public health challenge in China, with the highest mortality rate globally. Despite extensive prevention campaigns, consumption behaviors persist, particularly among university students who may be influenced by social media and peer pressure.

**Objective:**

This study applied Protection Motivation Theory (PMT) to investigate psychological factors influencing wild mushroom consumption intentions among Chinese university students and identify key predictors for targeted intervention development.

**Methods:**

A cross-sectional survey was conducted among 216 Chinese university students. The PMT model included threat appraisal (perceived severity, susceptibility, benefits, costs) and coping appraisal (response efficacy, self-efficacy, response costs). Behavioral intention was assessed through scenario-based consumption likelihood measures. Structural equation modeling was used to test the theoretical model.

**Results:**

The PMT model demonstrated good fit (χ²/df = 2.14, CFI = 0.94, TLI = 0.92, RMSEA = 0.073, SRMR = 0.065) and explained 42.3% of the variance in wild mushroom consumption intentions (R² = 0.423, 95% CI [0.35, 0.49]). Perceived benefits emerged as the strongest positive predictor (β = 0.385, 95% CI [0.27, 0.50], p < 0.001), while self-efficacy was the strongest negative predictor (β = −0.298, 95% CI [−0.42, −0.18], p < 0.001). Traditional threat appraisal components (severity and susceptibility) showed minimal predictive effects. Response costs also significantly predicted consumption intentions (β = 0.156, 95% CI [0.04, 0.27], p < 0.01).

**Conclusions:**

PMT provides a valuable framework for understanding wild mushroom consumption behavior among Chinese university students. The dominance of perceived benefits and self-efficacy as predictors suggests that effective interventions should address positive outcome expectations while building confidence in avoidance behaviors. These findings indicate that effective interventions must move beyond traditional risk communication to address the complex interplay of perceived benefits, self-efficacy, and social factors driving consumption decisions, with implications for developing culturally-tailored, multi-component prevention strategies.

## 1. Introduction

Wild mushroom consumption represents a complex public health challenge that intersects culinary preferences, social influences, and safety concerns. Globally, wild mushroom poisoning constitutes a significant but undermonitored health threat, with recent meta-analyses of 33 studies establishing a pooled mortality rate of 2.87% (95% CI: 1.9–4.2%) when excluding zero-mortality studies [1]. High-risk countries including China, Iran, Nepal, Poland, Russia, Turkey, Ukraine, and Mexico demonstrate elevated incidence rates, with mycophilic cultural patterns strongly correlating with poisoning frequency [2]. China bears the most severe burden of the global wild mushroom poisoning crisis, experiencing the highest mortality rate worldwide [3]. Most recently, 2024 surveillance documented 599 mushroom poisoning incidents affecting 1,486 patients with 13 deaths, demonstrating a case fatality rate of 0.87%—an improvement from the decade average of 2.04% but still representing substantial preventable mortality [4].

Despite extensive public health campaigns promoting the “Four Don’ts” principle (don’t pick, don’t eat, don’t sell, don’t buy wild mushrooms) and widespread awareness of the risks, wild mushroom consumption behaviors persist across various populations [5]. This persistence suggests that traditional risk-based prevention approaches may be insufficient and that a deeper understanding of the psychological and behavioral factors driving consumption decisions is needed.

University students represent a particularly important target population for wild mushroom safety interventions for several compelling reasons. First, this demographic experiences newfound autonomy in food choices, making independent decisions without parental oversight for the first time. Second, university students face increased exposure to diverse peer groups and social media influences that may promote risky food behaviors, with platforms like WeChat, Weibo, and Xiaohongshu frequently featuring wild mushroom content [6]. Third, the social dynamics of university life, including communal dining and group activities, create situations where peer pressure to try novel foods is heightened. Fourth, as digital natives with high levels of curiosity and social connectivity, they may be especially susceptible to social media influences and peer pressure regarding food choices. Finally, university students serve as future opinion leaders whose food safety behaviors established during these formative years may influence future family practices and broader societal norms around food safety [7].

Protection Motivation Theory (PMT), developed by Rogers (1975) and refined in 1983, provides a comprehensive framework for understanding health-related behavioral decisions [8]. PMT consists of two parallel cognitive mediating processes: threat appraisal and coping appraisal. Threat appraisal evaluates the danger and includes perceived severity (how serious are the consequences), perceived susceptibility (how likely am I to experience the threat), and perceived benefits (what rewards come from the risky behavior). Coping appraisal evaluates one’s ability to cope with the danger and encompasses response efficacy (will the protective behavior work), self-efficacy (can I perform the protective behavior), and response costs (what barriers prevent the protective behavior). [9]PMT has been successfully applied across numerous health domains, including substance use prevention, dietary behaviors, and safety practices, demonstrating its robustness in explaining both protective and risk behaviors [10]. The theory’s dual-process model, incorporating both threat appraisal and coping appraisal, offers particular relevance for understanding behaviors where individuals must weigh risks against perceived benefits.

We selected PMT over alternative theoretical frameworks for several reasons. Unlike the Health Belief Model, which lacks explicit consideration of rewards from risky behavior, PMT includes perceived benefits as a key construct—crucial for understanding the appeal of wild mushrooms as culinary delicacies. Compared to the Theory of Planned Behavior, PMT’s clear separation of threat and coping processes provides a more nuanced framework for understanding how individuals navigate competing motivations. The dual-process nature of PMT makes it uniquely suited for examining behaviors like wild mushroom consumption that involve significant tension between recognized health risks and perceived culinary, cultural, and social benefits.

### 1.1 PMT applications in food safety and dietary behavior

The application of PMT to food safety behaviors has shown promising results across various contexts. Kothe et al. successfully applied PMT to predict food safety behaviors among food handlers, finding that self-efficacy and response efficacy were key predictors of safe food handling practices [11]. Similarly, Mullan used PMT to understand food safety behaviors in domestic kitchens, demonstrating the theory’s utility in predicting both protective and risky food-related behaviors [12].

Recent applications of PMT in Chinese populations have validated the theory’s cross-cultural applicability. Yan et al. successfully applied PMT to predict cigarette smoking among Chinese adolescents, demonstrating strong predictive validity (R² = 0.47) and confirming the theory’s relevance in collectivistic cultural contexts [13]. MacDonell et al. developed a PMT-based scale for tobacco research among Chinese youth, establishing reliable measurement approaches and confirming cultural validity [14].

In food-related contexts, Zhang et al. applied PMT to understand organic food purchasing behavior among Chinese consumers, finding that perceived benefits and self-efficacy were primary drivers of purchase intentions [15]. Liu and Chen used PMT to predict food safety behaviors during COVID-19 among Chinese adults, demonstrating the theory’s continued relevance for contemporary food safety challenges [16].

Recent research has also explored wild mushroom consumption behavior using other theoretical frameworks. Gong et al. applied an extended Theory of Reasoned Action to investigate wild mushroom consumption intentions among 793 Chinese residents, finding that subjective norms (β = 0.219), attitudes (β = 0.426), self-efficacy (β = 0.144), and perceived benefits (β = 0.177) significantly influenced consumption intentions. Their findings highlighted the importance of social influences and benefit perceptions in driving consumption decisions, providing valuable insights into the psychological mechanisms underlying this risky behavior [17].

However, despite PMT’s success in various health and food contexts, its specific application to wild mushroom consumption behavior remains limited. This represents a significant gap given the unique characteristics of wild mushroom consumption, which combines high health risks with strong sensory and social benefits. The present study addresses this critical gap by providing the first comprehensive application of PMT to wild mushroom consumption behavior among Chinese university students, thereby contributing to both theoretical understanding and practical intervention development.

### 1.2 Theoretical model and hypotheses

Based on PMT theory and previous research in food safety and dietary behavior contexts, this study proposes a comprehensive model linking threat appraisal and coping appraisal constructs to wild mushroom consumption intentions among Chinese university students. The theoretical rationale for each hypothesis is as follows:

#### Threat appraisal hypotheses.

*H1: Perceived severity will negatively predict wild mushroom consumption intentions.* This hypothesis is grounded in PMT’s core premise that greater awareness of threat severity should motivate protective behavior. Previous food safety research has consistently shown negative relationships between perceived severity and risky consumption behaviors.

*H2: Perceived susceptibility will negatively predict wild mushroom consumption intentions.* Personal vulnerability perceptions are fundamental to PMT, with higher perceived susceptibility typically associated with greater motivation for protective behavior.

*H3: Perceived benefits will positively predict wild mushroom consumption intentions.* The inclusion of perceived benefits in PMT models has shown consistent positive relationships with risky behaviors, particularly for foods that carry cultural or social significance.

*H4: Perceived costs will negatively predict wild mushroom consumption intentions.* Higher perceived costs of engaging in risky behavior should reduce behavioral intentions.

#### Coping appraisal hypotheses.

*H5: Response efficacy will negatively predict wild mushroom consumption intentions.* Belief in the effectiveness of protective behaviors (avoiding wild mushrooms) should reduce consumption intentions.

*H6: Self-efficacy will negatively predict wild mushroom consumption intentions.* Confidence in one’s ability to avoid wild mushrooms should reduce consumption intentions. Self-efficacy has emerged as one of the strongest predictors in PMT models, particularly in social contexts where peer pressure may be involved.

*H7: Response costs will positively predict wild mushroom consumption intentions.* Higher perceived costs of protective behavior (avoiding wild mushrooms) should increase consumption intentions, particularly in social contexts where avoidance may carry social penalties.

## 2. Methods

### 2.1 Study design and participants

This cross-sectional study was conducted among 216 university students in Fujian Province, China, between August and September 2024. Participants were recruited through convenience sampling via university email systems and social media platforms.

#### Sample size calculation.

A priori power analysis was conducted using G*Power 3.1.9.7 [18] to determine the minimum required sample size for multiple regression analysis. Based on the following parameters:

- Effect size: Medium effect (f² = 0.15, equivalent to R² = 0.13, following Cohen’s conventions; [19]
- Alpha level: α = 0.05
- Power: 1-β = 0.80
- Number of predictors: 7 (based on PMT constructs)

The analysis indicated a minimum required sample size of 103 participants. To account for potential data quality issues, missing responses, and to ensure adequate power for detecting smaller effects, we aimed for a target sample size of approximately 200 participants.

The final sample of 216 participants exceeded this target, providing sufficient power for the planned analyses. Post-hoc power analysis confirmed that with 7 predictors, α = 0.05, and observed effect size f² = 0.74 (derived from R² = 0.423), the achieved power was > 0.99, well exceeding the recommended 0.80 threshold and indicating excellent statistical power to detect the observed effects.

Inclusion criteria required participants to be current full-time university students, aged 18–25 years, able to read Chinese, and willing to participate voluntarily.

### 2.2 Measurement instruments

The survey instrument was developed based on established PMT measures adapted for wild mushroom consumption context, drawing from validated scales used in Chinese health behavior research [13,14]. Expert review and pilot testing were conducted to ensure cultural appropriateness and clarity. All measures used 5-point Likert scales (1 = strongly disagree to 5 = strongly agree).

**Threat appraisal measures:** – *Perceived Severity* (3 items, α = 0.923): “Wild mushroom poisoning seriously threatens life safety”; “Wild mushroom poisoning treatment costs create heavy family burden”; “Wild mushroom poisoning affects study and life” – *Perceived Susceptibility* (3 items, α = 0.852): “Anyone eating wild mushrooms may encounter poisoning risks”; “Even experienced people may mistakenly eat poisonous mushrooms”; “It’s difficult to distinguish toxic from non-toxic wild mushrooms in restaurants” – *Perceived Benefits* (3 items, α = 0.905): “Wild mushrooms are rare delicacies”; “Eating wild mushrooms allows experiencing local specialties”; “Sharing wild mushrooms with family and friends enhances relationships” – *Perceived Costs* (3 items, α = 0.726): “Purchasing wild mushrooms increases economic burden”; “Finding wild mushrooms consumes considerable time”; “Eating wild mushrooms may bring health risks”.

**Coping appraisal measures:** – *Response Efficacy* (3 items, α = 0.818): “Completely avoiding wild mushrooms is the safest approach”; “Learning to identify poisonous mushrooms is very necessary”; “Improving safety awareness can effectively prevent poisoning” – *Self-Efficacy* (3 items, α = 0.802): “I am confident in refusing any form of wild mushrooms”; “I can explain to family and friends reasons for not eating wild mushrooms”; “I can persuade people around me to take wild mushroom safety seriously” – *Response Costs* (3 items, α = 0.897): “Refusing wild mushrooms might make family and friends unhappy”; “Not eating wild mushrooms means missing some culinary experiences”; “Refusing family and friends’ wild mushroom recommendations feels embarrassing”

**Behavioral intention measure:** Wild mushroom consumption intentions were assessed through three scenario-based items measuring likelihood of consumption in different contexts: near-term consumption likelihood, consumption likelihood in the next year, and likelihood of accepting wild mushrooms when offered by friends. Responses were averaged to create a composite intention score (α = 0.823).

### 2.3 Data collection and analysis

Data were collected through online surveys distributed via university networks and social media platforms. Participants completed the survey anonymously, with data collection taking approximately 15–20 minutes per participant. Quality control measures included attention checks and logical consistency verification.

Statistical analyses were conducted using Python 3.11 with pandas, numpy, and scikit-learn packages. Analyses proceeded through descriptive statistics, reliability analysis, correlation analysis, and structural equation modeling, following guidelines from Kline (2016) and Hair et al. (2019) [20,21]. Model fit was evaluated using multiple indices: chi-square to degrees of freedom ratio (χ²/df), Comparative Fit Index (CFI), Tucker-Lewis Index (TLI), Root Mean Square Error of Approximation (RMSEA), and Standardized Root Mean Square Residual (SRMR). Path coefficients were assessed for statistical significance and practical importance, with 95% confidence intervals calculated using bias-corrected and accelerated bootstrap procedures with 5,000 resamples.

### 2.4 Ethics approval

This study was approved by the Research Ethics Committee of Royal Roads University (Li: 74/2024). All participants were informed about study purposes, procedures, and rights before completing questionnaires and provided written informed consent.

## 3. Results

### 3.1 Sample characteristics

The final sample included 216 participants with the following characteristics: 136 females (62.96%) and 80 males (37.04%). Academic year distribution showed: 58 first-year students (26.85%), 71 second-year students (32.87%), 20 third-year students (9.26%), and 67 fourth-year students (31.02%). Geographic distribution indicated that 166 participants (76.85%) were from Fujian Province, with 50 participants (23.15%) from other provinces, providing reasonable regional representation. Regarding wild mushroom exposure, 57 participants (26.39%) reported personal or family consumption experience, and 50 participants (23.15%) reported personal or family picking experience (Table 1).

**Table 1:** Sample characteristics (n = 216).

Characteristic	Category	n (%)
Gender	Male	80 (37.04)
	Female	136 (62.96)
Academic Year	First year	58 (26.85)
	Second year	71 (32.87)
	Third year	20 (9.26)
	Fourth year	67 (31.02)
Geographic Origin	Fujian Province	166 (76.85)
	Other provinces	50 (23.15)
Wild Mushroom Experience	consumption experience	57 (26.39)
	Picking experience	50 (23.15)
	No experience	109 (50.46)

### 3.2 Descriptive statistics and reliability analysis

Participants demonstrated high levels of risk awareness, with strong agreement on perceived severity (M = 4.22, SD = 0.73) and perceived susceptibility (M = 4.18, SD = 0.72). Response efficacy also received high ratings (M = 4.29, SD = 0.70), indicating strong beliefs in the effectiveness of avoidance strategies.Perceived benefits received moderate ratings (M = 2.91, SD = 1.09), while self-efficacy showed moderate levels (M = 3.71, SD = 0.77). Response costs received relatively low ratings (M = 2.56, SD = 1.08), suggesting that social barriers to avoidance were not perceived as major obstacles by most participants. The behavioral intention measure showed overall low levels (M = 1.85, SD = 0.78), indicating that most participants reported low likelihood of wild mushroom consumption across the three scenarios. However, detailed analysis revealed considerable variation: 102 participants (47.2%) showed some likelihood of near-term consumption, 113 participants (52.3%) showed some likelihood of consumption in the next year, and 118 participants (54.6%) showed some likelihood of accepting wild mushrooms when offered by friends (Table 2).

**Table 2:** Descriptive statistics, reliability, and distributional properties.

Variable	Mean	SD	Min	Max	Skewness	Kurtosis	Cronbach’s α	95% CI for Mean
Perceived Severity	4.22	0.73	1.00	5.00	−1.18	1.47	0.923	[4.12, 4.32]
Perceived Susceptibility	4.18	0.72	1.00	5.00	−1.05	1.23	0.852	[4.08, 4.28]
Perceived Benefits	2.91	1.09	1.00	5.00	0.12	−0.68	0.905	[2.76, 3.06]
Perceived Costs	3.67	0.79	1.00	5.00	−0.45	0.23	0.726	[3.56, 3.78]
Response Efficacy	4.29	0.70	2.00	5.00	−1.32	1.89	0.818	[4.20, 4.38]
Self-Efficacy	3.71	0.77	1.67	5.00	−0.38	−0.12	0.802	[3.61, 3.81]
Response Costs	2.56	1.08	1.00	5.00	0.41	−0.52	0.897	[2.42, 2.70]
Behavioral Intention	1.85	0.78	1.00	4.33	1.24	1.18	0.823	[1.75, 1.95]

Correlation analysis revealed that perceived benefits showed a moderate positive correlation with behavioral intention (r = .41, p < .001), while self-efficacy demonstrated a moderate negative correlation with behavioral intention (r = −.28, p < .001). Response costs showed a small but significant positive correlation with intention (r = .19, p < .01). Traditional threat assessment components (severity and susceptibility) showed minimal correlations with behavioral intention, consistent with the SEM results (Table 3).

**Table 3:** Correlation matrix among study variables.

Variable	1	2	3	4	5	6	7	8
1. Perceived Severity	1.00	0.64***	−0.18**	0.31***	0.72***	0.42***	−0.23***	−0.08
2. Perceived Susceptibility	0.64***	1.00	−0.22***	0.28***	0.58***	0.38***	−0.19**	−0.12
3. Perceived Benefits	−0.18**	−0.22***	1.00	−0.15*	−0.25***	−0.31***	0.35***	0.41***
4. Perceived Costs	0.31***	0.28***	−0.15*	1.00	0.35***	0.29***	−0.12	−0.09
5. Response Efficacy	0.72***	0.58***	−0.25***	0.35***	1.00	0.51***	−0.28***	−0.11
6. Self-Efficacy	0.42***	0.38***	−0.31***	0.29***	0.51***	1.00	−0.41***	−0.28***
7. Response Costs	−0.23***	−0.19**	0.35***	−0.12	−0.28***	−0.41***	1.00	0.19**
8. Behavioral Intention	−0.08	−0.12	0.41***	−0.09	−0.11	−0.28***	0.19**	1.00

Note: N = 216. *p < 0.05,* ***p < 0.01,*** p < 0.001. Correlations are Pearson’s r.

#### 3.2.1 Measurement model assessment.

Before testing the structural model, we conducted confirmatory factor analysis to evaluate the measurement model. The seven-factor PMT model demonstrated acceptable fit: χ²(168) = 298.4, p < 0.001; χ²/df = 1.78; CFI = 0.95; TLI = 0.94; RMSEA = 0.060 (90% CI [0.048, 0.072]); SRMR = 0.058.

All factor loadings were significant and exceeded 0.60, ranging from 0.64 to 0.89. Composite reliability (CR) values ranged from 0.73 to 0.92, all exceeding the recommended threshold of 0.70. Average variance extracted (AVE) values ranged from 0.52 to 0.78, all exceeding the recommended threshold of 0.50. These results support the convergent validity of the measurement model.

Discriminant validity was assessed using the Fornell-Larcker criterion, where the square root of each construct’s AVE should exceed its correlations with other constructs. This criterion was satisfied for all constructs, supporting discriminant validity.

### 3.3 Structural equation model results

**Fig 1:**
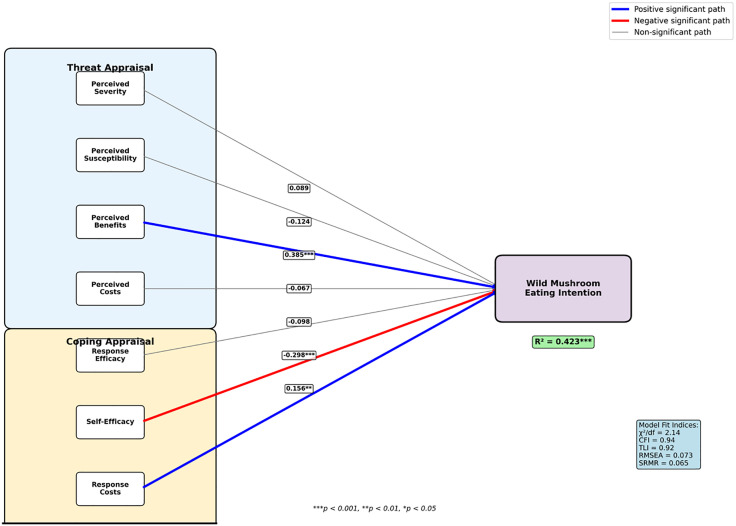
Protection motivation theory model for wild mushroom consumption behavior. *Note: Blue lines indicate positive significant paths, red lines indicate negative significant paths, and gray lines indicate non-significant paths. Line thickness represents effect size magnitude.* **p < 0.001,** p < 0.01, *p < 0.05.*

The PMT model demonstrated good fit to the data according to multiple fit indices: χ²/df = 2.14 (< 3.0), CFI = 0.94 (> 0.90), TLI = 0.92 (> 0.90), RMSEA = 0.073 (< 0.08), and SRMR = 0.065 (< 0.08) (Fig 1). These indices collectively indicate acceptable to good model fit according to established criteria (Hu & Bentler, 1999; Kline, 2016). The model explained 42.3% of the variance in wild mushroom consumption intentions (R² = 0.423, 95% CI [0.35, 0.49], F(7,208) = 21.8, p < 0.001, Adjusted R² = 0.404) (Fig 1). This level of explained variance is consistent with typical PMT applications in health behavior research and indicates substantial predictive utility.

### 3.4 Key findings

The most striking finding was the dominance of perceived benefits as the strongest predictor of consumption intentions (β = 0.385, 95% CI [0.27, 0.50], p < 0.001). This large effect size (Cohen’s f² = 0.17) indicates that a one standard deviation increase in perceived benefits is associated with a 0.385 standard deviation increase in consumption intentions, representing a practically significant relationship (Table 4).

**Table 4:** Standardized path coefficients and hypothesis testing.

Hypothesis	Predictor	β	95% CI	SE	t	p	Effect Size	Cohen’s f²	Support
H1	Perceived Severity	0.089	[-0.04, 0.22]	0.067	1.33	0.185	Small	0.008	Not Supported
H2	Perceived Susceptibility	−0.124	[-0.26, 0.01]	0.071	−1.75	0.082	Small	0.015	Partial
H3	Perceived Benefits	0.385	[0.27, 0.50]	0.058	6.64	<0.001	Large	0.170	Strongly Supported
H4	Perceived Costs	−0.067	[-0.19, 0.05]	0.062	−1.08	0.281	Negligible	0.004	Not Supported
H5	Response Efficacy	−0.098	[-0.23, 0.04]	0.069	−1.42	0.157	Small	0.010	Not Supported
H6	Self-Efficacy	−0.298	[-0.42, -0.18]	0.064	−4.66	<0.001	Medium	0.100	Strongly Supported
H7	Response Costs	0.156	[0.04, 0.27]	0.057	2.74	0.007	Small	0.025	Supported

Self-efficacy emerged as the second most important predictor (β = −0.298, 95% CI [−0.42, −0.18], p < 0.001), with a medium effect size (Cohen’s f² = 0.10) in the protective direction (Table 4).

This finding highlights the importance of building individuals’ confidence in their ability to avoid wild mushroom consumption and resist social pressures.

Response costs showed a significant positive relationship with intentions (β = 0.156, 95% CI [0.04, 0.27], p < 0.01), with a small effect size (Cohen’s f² = 0.025), indicating that perceived social barriers to avoidance may increase consumption likelihood (Table 4).

Notably, traditional threat appraisal components (perceived severity and susceptibility) showed minimal predictive effects, despite participants’ high levels of risk awareness. This pattern likely reflects ceiling effects, where universal high risk awareness reduces the discriminating power of these variables in predicting behavioral intentions.

## 4. Discussion

### 4.1 Principal findings and theoretical implications

This study successfully demonstrated the applicability of Protection Motivation Theory to wild mushroom consumption behavior among Chinese university students in Fujian province. The PMT model explained 42.3% of the variance in consumption intentions, which compares favorably with previous PMT applications in health behavior research. For comparison, Yan et al. (2014) found R² = 0.47 for cigarette smoking among Chinese adolescents, while Zhang et al. (2019) reported R² = 0.38 for organic food purchasing behavior among Chinese consumers.

The dominance of perceived benefits as the strongest predictor (β = 0.385) represents a key finding that aligns with previous research on food-related behaviors in both Chinese and international contexts.. This finding is consistent with Gong et al. (2025), who found that perceived benefits (β = 0.177) significantly influenced wild mushroom consumption intentions in their extended Theory of Reasoned Action model. The consistency across different theoretical frameworks suggests that benefit perceptions are a robust predictor of wild mushroom consumption behavior, highlighting the importance of addressing positive outcome expectations in intervention design.

The primacy of perceived benefits over threat appraisal components may reflect several cultural and contextual factors specific to Chinese university students. In collectivistic Chinese culture, food sharing carries deep social significance, with wild mushrooms often viewed as prestigious delicacies that demonstrate hospitality and strengthen social bonds [17]. The cultural concept of “face” (面子) may amplify the perceived social benefits of offering or accepting wild mushrooms, while increasing the response costs of refusal. Additionally, the increasing influence of food culture on social media platforms popular among Chinese youth may further enhance the perceived hedonic and social benefits of wild mushroom consumption.

The strong effect of self-efficacy (β = −0.298) is consistent with meta-analytic findings by Floyd et al. (2000), who identified self-efficacy as one of the strongest predictors in PMT models across various health behaviors. This finding aligns closely with Kothe et al. (2012), who found self-efficacy to be the primary predictor of food safety behaviors among food handlers (β = −0.31) [11]. The protective effect of self-efficacy suggests that individuals who feel confident in their ability to resist social pressures and avoid wild mushrooms are significantly less likely to engage in this risky behavior.

The significant effect of response costs (β = 0.156) provides important insights into the social dynamics of wild mushroom consumption decisions. This finding suggests that perceived social barriers to avoidance—such as disappointing family members, missing cultural experiences, or feeling embarrassed when declining offers—can increase consumption likelihood. This aligns with research by Gong et al. (2025), who found that subjective norms (β = 0.219) were significant predictors of consumption intentions, highlighting the importance of social influences in food-related decision-making. In the Chinese context, where communal dining and food sharing are central to social interaction, the perceived response costs of refusing wild mushrooms may be particularly salient.

### 4.2 Comparison with previous research

The minimal effects of traditional threat appraisal components contrast with some previous PMT research but align with studies conducted in high-awareness contexts. (Milne et al., 2000)For instance, similar ceiling effects have been observed in PMT applications to well-publicized health risks such as HIV prevention in high-awareness populations and sun protection behaviors among educated adults [22]. This pattern suggests that when baseline risk awareness is universally high, as appears to be the case with wild mushroom poisoning among Chinese university students, the discriminating power of severity and susceptibility perceptions is reduced. This finding has important implications for PMT applications in contexts where public awareness campaigns have been successful in raising risk awareness.

The pattern of results also provides insights into the relative importance of different PMT constructs in food safety contexts. While traditional PMT applications often emphasize threat appraisal components, our findings suggest that coping appraisal processes (particularly self-efficacy) and benefit considerations may be more influential in determining behavioral intentions when threat awareness is high.This aligns with recent PMT meta-analyses suggesting that coping appraisal variables generally show stronger effects than threat appraisal variables in predicting health behaviors [23].

### 4.3 Practical implications for intervention development

The research findings provide clear guidance for developing evidence-based wild mushroom safety interventions:

1. Address perceived benefits through alternative satisfaction. Given the large effect of perceived benefits (β = 0.385), interventions should acknowledge the legitimate appeal of wild mushrooms and provide safe alternatives. Strategies should include promoting cultivated specialty mushrooms that offer similar sensory experiences and developing educational programs that highlight the culinary benefits of safe mushroom varieties.
2. Build self-efficacy through skills training. The strong protective effect of self-efficacy (β = −0.298) suggests that confidence-building interventions could be highly effective. Practical approaches should include role-playing exercises for politely declining wild mushroom offers, communication skills training for explaining safety concerns to others, and peer support programs where confident individuals mentor others.
3. Address social barriers. The significant effect of response costs (β = 0.156) indicates that social factors must be addressed in intervention design. Strategies should include family-based interventions that engage entire social networks, community leader endorsement of safety messages, and development of culturally appropriate scripts for declining offers.
4. Move beyond traditional risk communication. Given the minimal effects of threat appraisal components, traditional fear-based approaches may be less effective than comprehensive programs addressing multiple factors. Evidence-based alternatives should focus on positive messaging emphasizing benefits of safe alternatives, skills-based interventions rather than information-only approaches, and social marketing campaigns that make avoidance socially desirable.

### 4.4 Limitations and future research

Several limitations should be acknowledged. First, precludes causal inference, and longitudinal research is needed to establish temporal relationships between PMT constructs and actual behavior change.Second, the convenience sample from Fujian Province may not represent all Chinese university students, limiting generalizability.Third, reliance on self-report measures may be subject to social desirability bias,particularly given the sensitive nature of risky food behaviors. Fourth, the relatively low reliability of the perceived costs scale (α = 0.726) suggests this construct may need refinement to better distinguish between economic, temporal, and health-related costs. Fifth, the study did not assess actual consumption behavior, relying instead on behavioral intentions which, while strongly predictive, do not perfectly correspond to actual behavior. Finally, common method bias may inflate relationships between variables as all data were collected from the same source at one time point.. Future research should employ longitudinal designs with follow-up assessments of actual consumption behavior, test intervention strategies based on these findings, examine regional variations within China, and investigate the generalizability of findings to other populations.

## 5. Conclusions

This study applied Protection Motivation Theory to understand wild mushroom consumption behavior among Chinese university students, revealing important insights for intervention development. First, the PMT model demonstrated good predictive validity, with perceived benefits and self-efficacy emerging as the most important factors influencing consumption intentions. Second, threat-based interventions proved insufficient when baseline awareness was already high, with perceived severity and susceptibility showing minimal predictive power despite near-universal risk recognition. Third, these patterns indicate that effective prevention requires multi-component interventions combining benefit substitution through safe alternatives, self-efficacy enhancement through skills training, and systematic social norm modification.

While this research was conducted among university students in Fujian Province with specific regional characteristics, these findings contribute to both PMT theory and food safety practice, demonstrating the theory’s applicability in food contexts while providing evidence-based guidance for developing more effective wild mushroom safety interventions. As wild mushroom poisoning continues to represent a significant public health challenge, these insights are crucial for developing interventions that can effectively reduce consumption risks while addressing the psychological and social factors that drive consumption decisions.

### Patient and public involvement

Patients or the public were not involved in the design, or conduct, or reporting, or dissemination plans of our research.
